# The Book World of Medicine and Science

**Published:** 1906-03-17

**Authors:** 


### The Book World of Medicine and Science.

An Essay on the Treatment and Cure of Pulmonary Consumption. By George Bodington, of Sutton Coldfield, a.d. 1840. Reprinted, with a Preface by Dr. Arthur E. Bodington, pp. 60. (Lichfield: Lomax's Successors. 1906.)

George Bodington was a pioneer, who, although unrecognised during his own life, has been justified by time; and this reprint of his original monograph is most welcome. The full title of Bodington's work exemplifies much of the nature of the man and his methods: "An Essay on the Treatment and Cure of Pulmonary Consumption, on principles natural, rational, and successful: with suggestions for an improved plan of treatment of the disease amongst the lower classes of society; and a relation of several successive cases restored from the last stage of consumption to a good state of health." By his own generation, Bodington's methods were derided and his work abused —and yet to-day throughout the civilised world he is honoured as one who alone saw the truth while other minds were blunted by prejudice and traditional teaching. Dr. Arthur E. Bodington, in rendering available for all the epoch-marking work of his now famous grandfather has earned the gratitude of every intelligent student and practitioner of medicine.

The Principles of Clinical Pathology. By Dr. Ludolf Krehl, Professor and Director of the Medical Clinic in Strasburg. Translated from the third German edition by Dr. Walter Hewlett, of San Francisco. (J. B. Lippincott Company, Philadelphia and London. 1905. Price 21s. net.)

This is the first appearance of this book in the English language, though it has already run through several editions in the German. The author deals with pathology from the clinical point of view, and takes up special subjects such as metabolism, nutrition, fever, which are scarcely touched by the ordinary text-books of general pathology and for a description of the pathology of which we are dependent on the short and often unsatisfactory accounts given in the text-books on medicine. There are chapters on the heart, blood-vessels, and lymph, including the subjects of œdema, infection, and immunity, etc.—all matters of the greatest interest to the clinician. Pathological anatomy does not enter into the subject-matter of the book, pathology being dealt with from the physiological standpoint entirely. In the chapter on infection and immunity, Ehrlich's "side-chain" theory of immunity might have been described at greater length with advantage, and unfortunately no mention is made of Professor Wright's work on "Opsonins." The purpose of the book, as the author states in the preface, "is to contribute to our knowledge of and to awaken interest in the theory of disease processes." We recommend this book to the attention of the advanced student and to the practitioner who desires to keep his knowledge of the processes of disease abreast of the recent advances that have been made in this direction.

Surgical Diseases of the Dog and Cat, with chapters on Anæsthetics and Obstetrics. By Frederick T. G. Hobday, F.R.C.V.S., F.R.S.E. (London: Baillière, Tindall and Cox. 1906. Second edition. Pp. 366.)

We gladly welcome a second edition of Professor Hobday's Canine and Feline Surgery, for it is thoroughly practical and is well illustrated. The book shows the strides taken of late years by the veterinary profession towards making its handicraft a science. Many years ago surgery made a similar advance, and it has now come to dominate medicine. Professor Hobday has brought to his book a very thorough knowledge of bacteriology and comparative pathology, and with it he unites a familiarity with the details of practice as it affects dogs and cats, for he is well known as the consulting surgeon to the British Bulldog Club and to the Fox Terrier Club, as well as to the Ladies' Kennel Association. It has been his aim throughout the book to introduce the latest surgical methods, modified to suit the special needs of animals, and he expresses his ideas so clearly and concisely as to render his work of the utmost value. It is pleasant to notice that Professor Hobday recognises the intimate connection between the veterinary and the medical professions, because he gracefully dedicates this edition to Mr. D'Arcy Power, from whom he professes to have received kindness.

Genito-Urinary Surgery and Venereal Diseases. By J. William White, M.D., and Edward Martin, M.D. (J. B. Lippincott Company, Philadelphia and London. Sixth Edition. Pp. 1,092, with 300 engravings and 14 coloured plates. Price 21s. net.)

This is an excellent book, and the appearance of six editions in rapid succession shows that its merits are well known and appreciated. In this edition the work has been brought up to date, and embodies the many small but not unimportant improvements which have recently been made in the field of genito-urinary surgery; and especially noteworthy in this respect are the chapters on examination of the urinary organs, and injuries and diseases of the prostate. The subjects of syphilis and gonorrhœa are comprehensively dealt with. The work is essentially a practical one, and will be a valuable book of reference for both the general practitioner and the specialist.

The Writers' and Artists' Year-book, 1906. (London: Adam and Charles Black, Soho Square, W. Price 1s.)

This year-book contains lists of newspapers and magazines, English and American publishers and literary agents, together with some details respecting the correction of proofs and the dispatch of MSS., and should prove exceedingly useful to the ever-increasing army of writers and artists. We notice one or two important omissions which we think should be rectified in future issues.

